# Supplementary material for: Mechanical Properties of a Solvated Biomolecule: RGD (1FUV) Peptide
Source: Int J Mol Sci. 2024 Sep 21;25(18):10164. doi: 10.3390/ijms251810164 (PMC11432424; doi:10.3390/ijms251810164)
Supplement: Supplementary file 1 [file ijms-25-10164-s001.zip › ijms-3179887-supplementary.pdf]

Supplementary Materials for

Mechanical properties of a solvated biomolecule: RGD (1FUV) peptide

Puja Adhikari [1], Bahaa Jawad [1,2] and Wai-Yim Ching [1*]

[1] Department of Physics and Astronomy, University of Missouri-Kansas City, Kansas City, MO 64110, USA. (PA: paz67@umkc.edu)

[2] Department of Applied Sciences, University of Technology, Baghdad 10066, Iraq (BJ: bahaa.a.jawad@uotechnology.edu.iq)

* Correspondence: WYC: chingw@umkc.edu

Table S1: Five sets of mechanical properties of solvated RGD (1FUV). *K*, *G*, *E* are in unit of GPa.

Strain%	*K1*	*K2*	*K3*	*K4*	*K5*	*G1*	*G2*	*G3*	*G4*	*G5*
0.25	6.61	6.69	6.56	6.64	6.43	3.50	3.69	3.43	3.66	3.59
0.50	6.24	6.30	6.38	6.36	6.22	3.60	3.56	3.62	3.55	3.57
0.75	6.21	6.26	6.18	6.31	6.36	3.54	3.60	3.56	3.50	3.61
1.00	6.17	6.13	6.03	6.20	6.07	3.51	3.41	3.54	3.50	3.54
1.25	5.96	6.16	6.00	6.06	6.12	3.50	3.52	3.49	3.48	3.45
1.50	5.92	6.06	6.05	6.01	6.11	3.47	3.49	3.45	3.44	3.50
1.75	6.07	5.82	5.94	5.79	5.76	3.47	3.43	3.43	3.47	3.40
2.00	5.84	5.85	5.97	6.00	5.95	3.41	3.41	3.45	3.47	3.44
2.25	6.01	5.78	5.93	5.75	5.81	3.44	3.40	3.44	3.41	3.40
2.50	5.78	5.85	5.98	5.89	5.88	3.38	3.43	3.45	3.44	3.43
**Strain%**	***E1***	***E2***	***E3***	***E4***	***E5***	***η1***	***η2***	***η3***	***η4***	***η5***
0.25	8.92	9.34	8.77	9.27	9.08	0.2751	0.2672	0.2772	0.2671	0.2646
0.50	9.06	8.99	9.12	8.99	9.00	0.2581	0.2624	0.2618	0.2643	0.2589
0.75	8.93	9.07	8.95	8.87	9.10	0.2603	0.2587	0.2585	0.2657	0.2613
1.00	8.86	8.62	8.88	8.85	8.90	0.2607	0.2657	0.2545	0.2623	0.2556
1.25	8.79	8.86	8.77	8.76	8.71	0.2543	0.2602	0.2562	0.2590	0.2627
1.50	8.71	8.78	8.71	8.67	8.82	0.2550	0.2584	0.2601	0.2595	0.2595
1.75	8.75	8.60	8.64	8.67	8.53	0.2598	0.2539	0.2578	0.2505	0.2532
2.00	8.56	8.56	8.68	8.74	8.66	0.2557	0.2562	0.2579	0.2572	0.2574
2.25	8.66	8.53	8.64	8.53	8.53	0.2597	0.2542	0.2571	0.2526	0.2553
2.50	8.48	8.61	8.69	8.63	8.61	0.2553	0.2549	0.2581	0.2560	0.2560

Table S2. Eleven amino acid residues with total AABP and respective contribution from NN, NL and from HB (Only including inter-AA interaction and in unit of e^-).

AA	Total AABP	NN	NL	AABP from HB	No. of NL AAs
**Ala1**	0.5844	0.4021	0.1823	0.2122	4
**Cys2**	0.9618	0.8245	0.1373	0.1669	4
**Asp3**	0.8254	0.8251	0.0003	0.0496	1
**Cys4**	0.9514	0.8572	0.0942	0.1342	2
**Arg5**	1.1100	0.8964	0.2136	0.2559	2
**Gly6**	0.8334	0.8318	0.0016	0.0467	2
**Asp7**	0.9122	0.7618	0.1504	0.2046	2
**Cys8**	0.9449	0.7787	0.1662	0.2293	6
**Phe9**	0.9176	0.7763	0.1413	0.2096	5
**Cys10**	0.8107	0.7815	0.0292	0.0587	3
**Gly11**	0.4308	0.4120	0.0188	0.0383	1

Table S3. Interaction details of eleven amino acid residues with water molecules.

AA	Total AA-H_2O BO	AA-H_2O HB
**Ala1**	0.2008	0.1942
**Cys2**	0.0002	-
**Asp3**	0.3805	0.3636
**Cys4**	0.1491	0.1233
**Arg5**	0.1354	0.1209
**Gly6**	0.1474	0.1400
**Asp7**	0.1927	0.1799
**Cys8**	0.0049	0.0002
**Phe9**	0.0146	0.0128
**Cys10**	0.1151	0.1070
**Gly11**	0.1938	0.1859

Optimized solvated 1FUV structure coordinates (POSCAR)

```
POSCAR
System   N14C42O171H369S4
   1.0000000000000000
    23.6709999999999994    0.0000000000000000    0.0000000000000000
     0.0000000000000000   21.3610000000000007    0.0000000000000000
     0.0000000000000000    0.0000000000000000   15.3670000000000009
   N    C    O    H    S
    14    42   171   369     4
Direct
  0.7043386261200290  0.3995727962001336  0.3588616950916858
  0.6753225861740465  0.5200954361222307  0.5218985973907065
  0.6494888951891074  0.6511331552663274  0.5332164457363474
  0.5460806207627803  0.7285095823109562  0.5455724255610291
```

0.4201792188877714	0.6934326617719132	0.5959180438712995
0.1946065475382183	0.7526780834354178	0.5934935933825026
0.1374555689083252	0.6817325201088316	0.5176039068561599
0.0986783487453337	0.7466183874601275	0.6230923813552572
0.3283627216789270	0.5670827251860677	0.6621912216890858
0.3081050090058867	0.5156851203053748	0.4927744757557175
0.4119920965851747	0.5067177397156094	0.4100335540491875
0.5065467887947532	0.3887210307084443	0.4448617752282530
0.6023417820494454	0.3312411760923282	0.6117778841231624
0.6097298079651630	0.2115898250534355	0.6745569379633644
0.7247166607309012	0.4474495490055044	0.4226288905092219
0.6724141731261811	0.4868695000123540	0.4463503755003469
0.7724509932042829	0.4870957680079808	0.3848928908101997
0.6246299850114916	0.5414395162880964	0.5670022855883320
0.6214438472787905	0.6119444341557081	0.5862961541398027
0.6119163358598833	0.5030729029768309	0.6491388632919673
0.6498972913621114	0.7189730619615775	0.5473674872382960
0.5950880714993765	0.7493486237215520	0.5097790997875820
0.7018607228826254	0.7488778416525624	0.5059128259835285
0.7595813967301436	0.7284160835636931	0.5395454181362244
0.4913330591396192	0.7472434636620832	0.5103729553474449
0.4460626314811829	0.7484036401706419	0.5842097539151946
0.4705796865116679	0.7063881398290056	0.4333147789358342
0.3732893512220803	0.6730042663013809	0.6505669942484912
0.3691848421110462	0.6022063163827208	0.6262953718849072
0.3182379525249063	0.7113090624728329	0.6385423344600969
0.2900331383064736	0.7059726503370911	0.5492473030084651
0.2420725294699046	0.7531546031892111	0.5321035764904837
0.1437395958774393	0.7266715371079524	0.5781062682168511
0.3275072831141351	0.4991717928040313	0.6489657166213509
0.3260348704435229	0.4757307409104155	0.5550072715747318
0.3073876399866762	0.4996179100392778	0.4011570588540572
0.3671663349819521	0.4842432313998722	0.3644386341068063
0.2802774804101527	0.5530664820500031	0.3481541752317162
0.2203294301613846	0.5687765642296738	0.3805725773702680
0.4701859022259902	0.4909511440533695	0.3884814402636060
0.4950059540360713	0.4496338670870639	0.4626196435696271
0.5080886685940220	0.5491840709574921	0.3852306382991677
0.5217553197804801	0.3460581706994831	0.5147802673231644
0.5861964868569032	0.3383123114893307	0.5265576538246747
0.4916102453888067	0.2818103792960381	0.5038991045035861
0.5041500897123438	0.2458422278747010	0.4215456681031438
0.5478841139046745	0.2014292599231725	0.4184860963587170
0.4704898002492541	0.2541799422329427	0.3471248485625331
0.5575640281465127	0.1660868256745609	0.3435869801074025
0.4801956287336531	0.2191907603651116	0.2716790320275669
0.5236415392273943	0.1749005713404978	0.2699896295258909
0.6577642167424845	0.3100795263873653	0.6404194905268603
0.6595894097524468	0.2400304066621028	0.6683743295692505
0.6813284047089392	0.3494197402925089	0.7163359576584123
0.5970057514587727	0.1501819263394845	0.7095730890313323

0.5324214397098630	0.1492170758953059	0.7254823735973913
0.6307798126027190	0.4870191708357216	0.3975754285651098
0.5898434411015622	0.6308394121093198	0.6458245440482452
0.5971331673458440	0.7888549041367930	0.4506806439776266
0.8017021371473823	0.7635266441063047	0.5200354532121397
0.7658316730188947	0.6782474462097875	0.5821868058995913
0.4364992475481380	0.7974590336775064	0.6258696470582122
0.4037859584869707	0.5799542347616525	0.5728648360926744
0.3425477204242114	0.4215987405635053	0.5381348385717581
0.3714288666454547	0.4536582359122279	0.2963801031268511
0.1794674362935985	0.5601046145366544	0.3297766324728782
0.2162028317659170	0.5892216998870605	0.4591698123820393
0.5030155991728005	0.4728440268722373	0.5354677145998912
0.6201085360437968	0.3363068692927065	0.4653052425700177
0.7060190808627187	0.2146673352512326	0.6845135664398371
0.5120360432437484	0.0994552734014275	0.7556698482361192
0.5054128729452058	0.1990186471455798	0.7056146542767812
0.7326598494745199	0.5782364249035657	0.2197698192326907
0.9322715416570199	0.6028745990897922	0.2174904147295094
0.8846975156946280	0.0482226016765836	0.5159691513242300
0.4285868143599023	0.0111926028032685	0.5559661649549794
0.1165865625505408	0.6684431425949262	0.9629886182810447
0.0509404194150285	0.9444508999912099	0.9626774197191218
0.1218734974477194	0.5160395942906817	0.7208362587494146
0.0623219494557526	0.1376821505535009	0.3238602041091401
0.5736573449178384	0.8651373411925192	0.9710721665221854
0.8838032827922914	0.0905916767846151	0.8326379740200885
0.6634310780664363	0.9467398081123569	0.9950985262915817
0.2205829643986605	0.0644220126146772	0.0076746835812282
0.4294453347325354	0.9768290200706535	0.2446210243086560
0.9984840169220375	0.1721454707983438	0.0419967819785948
0.3610090589669505	0.8742273411261805	0.2721802167097749
0.2980305772320400	0.0578382704974099	0.7390042063708732
0.8328361727117141	0.9938886626693406	0.9094130517082258
0.7465504588329398	0.8941358189339829	0.3872680866352706
0.9975606350988310	0.9770827109635882	0.1199057281264780
0.8815013709476166	0.8146525064406489	0.9161771852099130
0.3457248259958071	0.2865866442918844	0.2661740960291309
0.9747391902439118	0.0024118586549460	0.8364031953062501
0.3717431489727833	0.9075027622809786	0.5843388103716941
0.9625074977557588	0.8673697678495996	0.6697421403951503
0.2701999279856782	0.1890920176874456	0.3272543443188696
0.0682698737876905	0.4363361636625985	0.8232311149397081
0.3834552940819282	0.0147165286987875	0.0870881454863566
0.5732821766199536	0.7171730793863837	0.0895941303566338
0.2115243410221207	0.2986803589234530	0.3104568590629164
0.1517693276028556	0.2516471800392219	0.0748757973484137
0.8401174769775063	0.2316013850888562	0.0512696279841115
0.3782604935804820	0.8984715297535931	0.9988088999259960
0.2937264780082957	0.2280551722728566	0.5068216543597442
0.1015649207570995	0.3190581139850054	0.3140634314593154

0.5405222104988920	0.9487333702488121	0.2497646079360335
0.6518397948730537	0.7685162642178218	0.9726893770846383
0.7298847954951920	0.8019713882879552	0.2513909030326542
0.9660944942829751	0.7397274251653478	0.6749649707756589
0.0505232541961092	0.2274239644090543	0.5657788947109402
0.0380103089195167	0.1592944389824925	0.8760470406408066
0.0917432260620116	0.1471706586646293	0.1484861385255301
0.8462339793100500	0.6653396072824485	0.0232240480479969
0.0591893390912954	0.8310003431433951	0.8916131397501880
0.8000708761773665	0.1296234472942617	0.2619791053781761
0.2103311848056417	0.6892144582241421	0.8497128643982891
0.9532414093834838	0.6842733297802692	0.0888768093358552
0.9307009451686298	0.3853217030558846	0.0073272140237108
0.8923515984848551	0.9059898353007841	0.7961168031280953
0.8342223187791883	0.5679447825180433	0.1405877050743414
0.9429788830787120	0.6673578247672156	0.3711826142938293
0.0163135195871442	0.0914371492428280	0.7213194423646373
0.1439970113864049	0.0316737295474285	0.1954972652757025
0.2800554410765809	0.4142320934565318	0.8937723823476619
0.1468671513037317	0.3502244203846830	0.7261700656978002
0.8423556839139513	0.8127860658143510	0.2191273721315822
0.2256868607817145	0.8144996622275503	0.8759278776305953
0.3660751008247268	0.1959881660707587	0.9247616905204759
0.9336350886182168	0.8742691398076822	0.3805625099166467
0.7168822771271924	0.8433216388879202	0.0793827730989379
0.9789649979651450	0.0538505376084821	0.3653642869360925
0.2409558056785349	0.3258163268792946	0.1492391922600043
0.8783973433496535	0.2380950060615794	0.5585134388872384
0.5151251802041994	0.8640787605734165	0.3729784997660231
0.2782420964064155	0.1234954372376637	0.8857270182281520
0.9605979061453747	0.1952143557022743	0.6597968985758570
0.4654033864393187	0.8260807185722064	0.9373368690233617
0.4041978604970917	0.0538602755891796	0.3828890091812024
0.3020554180266050	0.6227400865212547	0.8360607865524265
0.0763328256825401	0.5184074706820616	0.3742133308448926
0.3260744586404616	0.8640860471252101	0.8474673524696417
0.0555167010625419	0.9738044989582013	0.2726608297584868
0.9540156426593220	0.2824788964705510	0.1057460844575629
0.2171241600444759	0.0739550356395534	0.3215434682972622
0.1736500439597473	0.4899614824306518	0.5656713081296058
0.7831909642867713	0.0176486176956127	0.1892613124430435
0.6170003107236267	0.6281153002812632	0.1908014315199100
0.8941727108519193	0.3635236836930613	0.5656686925664995
0.1818835217313759	0.1138749032783344	0.7310416391859309
0.9370558261188060	0.0769607202951335	0.1132121465814678
0.1022809502378583	0.1125509846097734	0.6058278903056543
0.6960974761425393	0.6820672732746004	0.3036377799849180
0.7987283308237685	0.8866665000479049	0.5458391543153785
0.7747779216715419	0.0871004185956092	0.4264053879219534
0.0638323145007975	0.3264570834713806	0.1413763182996293
0.1130899144571717	0.7622743638119812	0.0785411177255319

0.8928091674389294	0.3269950926936274	0.2803996691982368
0.8524147265960376	0.4856489437344251	0.9995715452849650
0.2019808377064539	0.8456910602688085	0.0437314203512050
0.0345663980067075	0.8539022342372703	0.2950967809733127
0.0288935673293543	0.5300341420744117	0.2117038351618341
0.7935989303511359	0.9420529020571263	0.7046053267406610
0.9078673900989586	0.9246866533073429	0.5343841045706436
0.9887883119806671	0.6275374582177441	0.9403201829546929
0.3834296546768530	0.7317403598089349	0.1431847809741833
0.1837031862228107	0.4811530532241206	0.8838300894272614
0.8345353850749292	0.5728011649272367	0.7505180093584053
0.3051191971899961	0.2713979095521782	0.6803399733560875
0.3027037184961428	0.3358226693402962	0.4169422395321830
0.9683261350537240	0.2518780480016173	0.8195944141531848
0.3940445914151985	0.2068359254561292	0.7503520158541694
0.1840404092292461	0.2279071390556349	0.5328066593421994
0.8127914399912973	0.2581337398711581	0.7100550249208082
0.7588636190614161	0.2256271750887227	0.1729417554348803
0.7107192513192011	0.0073888898011728	0.3367750337837002
0.8393398637676796	0.9257679598277297	0.2973393026294978
0.3151732637612771	0.8949243332038124	0.4274683952709267
0.6191094886972243	0.0393663793483853	0.2573620134785132
0.2898659287254530	0.9312140779871511	0.7078173241987330
0.8854611544917954	0.5825751408200418	0.9061375428817903
0.0194854490483723	0.4400652055378575	0.0959195509148564
0.8788326214772192	0.7656329266231059	0.3758049795734264
0.4049470933377677	0.7814487067215247	0.8011832573230588
0.9963440813347998	0.4138621981319978	0.6184970464091876
0.2809330245775200	0.0982037625313265	0.5618499031297348
0.0284522995599251	0.2436827188225894	0.3952063113804722
0.8634295989139006	0.9836524069986793	0.0739502324696985
0.9868967777169516	0.4971588207459692	0.4801998982919531
0.3447841933149889	0.5173488440358490	0.9146920324922579
0.0005444834859224	0.7973561100007063	0.1328882195422092
0.0849041504165979	0.3459398869144310	0.5843828911905191
0.8769955050278605	0.6556129438588023	0.6347882412379604
0.9050809614455655	0.8605836516829776	0.0809327205111125
0.0449353028073781	0.5114156108093363	0.9601775816967816
0.9118569918057200	0.1241273644912752	0.2699774665233212
0.3114438763922097	0.8343207308065086	0.1161586829709846
0.1535238774048953	0.9621653474377464	0.0403152004189590
0.5523893584675946	0.8446605064318270	0.1430444022018250
0.8349561043597482	0.0652370435048047	0.6706831338977526
0.2137366055825740	0.2274405340717941	0.9181101591537431
0.4626572916928421	0.8012921106743544	0.2519077105353338
0.1944619040332780	0.8582845264801652	0.7146569448678830
0.1434771031755471	0.1233378019064247	0.4438669654715144
0.7771889554844746	0.3289636077358049	0.2761593290114522
0.2919190874294065	0.0756165386957548	0.1385958856651710
0.4701369293179118	0.7120404420139198	0.0242956840802317
0.9630277657594190	0.3770565324764956	0.8354663079378254

0.8647424441704639	0.2120916620002392	0.8692495836824088
0.6798775974727989	0.0449942565096809	0.1050809008113191
0.0781350704367522	0.8609452857855542	0.7159463896781000
0.2627144039882194	0.3829396218399704	0.7233924455408197
0.7226376757306259	0.9806162224340150	0.8552869089269057
0.9719398283404547	0.7447461136456807	0.8591456060893676
0.1202892942081643	0.4091376184979069	0.4386648041615887
0.9160849372064496	0.2280259114873466	0.3806841929319831
0.1454553982771729	0.5576814376718232	0.0163377685962043
0.7825233589775445	0.7630168484776584	0.9728524969550042
0.9083269847382468	0.4234051698775212	0.4080733489576572
0.4039960621316543	0.0864150046791069	0.6988962774375843
0.8554590051364436	0.3784088145054451	0.7342071642764493
0.9097297970932253	0.5829458321827516	0.4973789962650549
0.6653066182199379	0.1724340693520160	0.1048835842777237
0.1324060964164146	0.0975254492688961	0.8965091991194344
0.1988674533257241	0.2390473568929949	0.7355811706814056
0.2986322556458412	0.0244515357245392	0.4220879881295550
0.2947974040451634	0.3040099421272647	0.9797874858905489
0.7374393223372563	0.3717706003850984	0.3269583193953701
0.6811736089908420	0.4228638709525306	0.3122831433920415
0.6759540550266293	0.3699572091224394	0.3899041932749196
0.7390865628105714	0.4218681027407423	0.4807228080127032
0.8105016537698424	0.4588014755654774	0.3736886511790519
0.7833956272402799	0.5240808680602241	0.4317138938705204
0.7591116973846666	0.5101512574633766	0.3242836354130404
0.7099375928595083	0.5135346958198073	0.5602164016558029
0.5891374361985452	0.5323177713400273	0.5223284304551690
0.5782579022191694	0.5271915042831176	0.6852216708535833
0.5966148604439081	0.4564692916099319	0.6312773058589912
0.6749362696240563	0.6336737672353832	0.4857151902307708
0.6494222105028687	0.7262562650742761	0.6184015347120511
0.7013679343982232	0.7391631123516110	0.4354239355592798
0.6985837936097534	0.7999707841922765	0.5113792925729419
0.5495784786703356	0.6949405031837954	0.5934965541529077
0.4949799025137422	0.7960044770190488	0.4886006401772161
0.4553973063117165	0.6605437728457225	0.4551160092812890
0.4352717154343818	0.7302973990344942	0.4008972350222643
0.4344994634404690	0.6550287394455920	0.5620901497711653
0.3854920025443921	0.6766174914228928	0.7196925806486104
0.2886235555949162	0.6966691571858837	0.6898242464043850
0.3289843298641944	0.7603816903735646	0.6527595255954147
0.3214214681976361	0.7142305514161359	0.4974313176592199
0.2737679096416663	0.6583190084855921	0.5391807409280185
0.2592429484827445	0.8010434698756621	0.5325334747483212
0.2254293210479555	0.7451773183407279	0.4662102693401849
0.1954193497602076	0.7868277680239811	0.6410274904883302
0.1696371497109275	0.6529227081817119	0.4965758987850964
0.0977988428319598	0.6671359663900402	0.5034442881091835
0.0979850211052821	0.7883801898986079	0.6569846222306082
0.0610773368749771	0.7235870043241388	0.6208544306399371

0.3078687014599881	0.5851580381175489	0.7156939755820837
0.3650622757745003	0.4773478007919905	0.6782788729827718
0.2904431336852435	0.4790861405603875	0.6818766437303480
0.2853227807543310	0.5548074132742027	0.5091515555970569
0.2831117628356996	0.4562631744220247	0.3904470968805726
0.3063690972557502	0.5955877442466203	0.3537571534713920
0.2787075090677075	0.5391790715199019	0.2795995208938716
0.4051566322359512	0.5286593558396204	0.4686186598274602
0.4694105022793940	0.4657808822344121	0.3260687863936122
0.5048914307063628	0.5728224053157855	0.4485559787958747
0.5522202774994747	0.5351711610429067	0.3752866504797551
0.4994389145763381	0.3706554456159357	0.3846169913427497
0.5051491949410788	0.3677332466536144	0.5745674897938592
0.5015185316170976	0.2529600363859623	0.5612520820208352
0.4460951186315710	0.2920122632513583	0.5074807291548321
0.5737962628388570	0.1934207299119939	0.4762562632108945
0.4347590079553458	0.2865100335778984	0.3481337229410966
0.5904554186757786	0.1303568274255522	0.3418340771264933
0.4527927182454063	0.2258888902056783	0.2153868267094418
0.5303391309053447	0.1459330294142192	0.2123879187176671
0.5719490653227592	0.3367328727668025	0.6579665769085176
0.6859906732497285	0.3137772769958032	0.5838096024043523
0.6482018655535299	0.3621782385401906	0.7632108196976297
0.7132218059135368	0.3217899837907193	0.7511083748074264
0.5725743419745595	0.2355687416842950	0.6648002728990459
0.6080139436742509	0.1124318373138146	0.6639254734099311
0.6198427230172583	0.1413381858335873	0.7706352755945385
0.6979674708517687	0.5787516290726933	0.1850933648432655
0.7284436280769335	0.6177796630711211	0.2541583445081884
0.9626368134882041	0.5709235549104849	0.2150003594107932
0.8958260201245514	0.5842766265250176	0.1963488680030304
0.8927023786857143	0.0020025292858785	0.5191032059573292
0.8521262872015036	0.0536568065829609	0.4770103732456952
0.4245357940103940	0.0293888722688479	0.4973917563469963
0.4189983831514034	0.0441469804814706	0.5987806155768801
0.1308071704886288	0.6259046609304838	0.9857474564857516
0.0795264952069583	0.6589531487186091	0.9380140350799315
0.0896034195604507	0.9584042747152439	0.9804883419214473
0.0344509753194490	0.9739473159269526	0.9202770023467340
0.1535348124842900	0.5150092190355676	0.7618975033879897
0.1384067544377310	0.5052864900801365	0.6631054727110890
0.0938134719594406	0.1301050994122920	0.3664200928656984
0.0326436430771697	0.1064438513552390	0.3399455878398190
0.5995508676161856	0.9018390885292602	0.9722838659637875
0.5996710047301949	0.8290674353641897	0.9606277830462638
0.8709399745853951	0.0839404659022283	0.7717886215853240
0.8609546026224960	0.0595865091719084	0.8668544320649738
0.6839927437316042	0.9560647785826245	0.9391561533525956
0.6853536796211802	0.9134543602042112	0.0245563981119509
0.2443869148462767	0.0690336688668247	0.0618857520309871
0.2028166987796105	0.0224794224135009	0.0112358810488906

0.4045840510296522	0.9398222486681140	0.2558246712107497
0.4218298640804317	0.0062046440979816	0.2936483050727758
0.0323576473076845	0.1626651140509703	0.0782585699403925
0.0101516143502293	0.1651391878317241	0.9802074864638012
0.3903784557750012	0.8418068526735042	0.2756101202856569
0.3445579182768922	0.8791502115454881	0.3328182331009622
0.3399312752994248	0.0628736941010165	0.7296907208890920
0.2911864689483702	0.0799036762218480	0.7962643173987670
0.8438663343201432	0.9881093671003733	0.9724486197752373
0.8535792198373967	0.9622256279845283	0.8758314855058242
0.7592375137096681	0.8940374505611629	0.4490372854939359
0.7253124400385049	0.9338770265856752	0.3775954444564682
0.0182307434218529	0.9634750763334876	0.0674031562445753
0.0225501961736286	0.9738186498628800	0.1717848155006139
0.8440198117288180	0.7941177491706924	0.9231993921526189
0.9099177948682042	0.7826317115972053	0.8984498904993464
0.3255548185218333	0.2466356251874473	0.2755714776573513
0.3271758773863662	0.3055285977669668	0.2159234898860767
0.9501941508270949	0.9660527322967044	0.8238781624113539
0.9473248646117713	0.0364831044945149	0.8477462027511116
0.3988285202767695	0.8745076748807524	0.5998229978382505
0.3948232434114222	0.9469690332012455	0.5733655653846864
0.9455244642736784	0.8869813694554848	0.6166610446467706
0.9596841814067937	0.8207560567891490	0.6630600985586951
0.2813598340179062	0.1918289361259111	0.3891285475431110
0.2427807276259762	0.2241882299007446	0.3208392625671678
0.0306061790298106	0.4173193849029376	0.8122720009432280
0.0794373741036290	0.4644966267623947	0.7734267844259347
0.4035322898636389	0.0021793869253699	0.1409899956542016
0.3811190357010392	0.9764384757178770	0.0508369425547931
0.5719795641271557	0.7583955925257119	0.1188039397453659
0.5335069541738180	0.7116963889620872	0.0652496669356372
0.2318550233378054	0.3216794123607375	0.3565338544352801
0.1694182727172625	0.3045885439010796	0.3174121004768135
0.1658146442404941	0.2395649622442082	0.0164012943943327
0.1836305036178729	0.2751480613982613	0.1007608684890639
0.8442209538551488	0.2269013035284708	0.9877301477488505
0.8758424568872970	0.2489102591222062	0.0726739088926821
0.3545480843825538	0.8751155495737667	0.0415327179597279
0.3573896534248790	0.8943591033486378	0.9427644933692003
0.3019392349374848	0.2680204991041766	0.4750598038792789
0.2513147508593710	0.2291843056824965	0.5156697721888720
0.0736493068580742	0.2915589489698975	0.3457066718981133
0.0867767036661649	0.3244611261794879	0.2539393206811905
0.4997994106347373	0.9609101651713904	0.2424604964217326
0.5485362016284431	0.9158333804888333	0.2055325324625840
0.6715210390626510	0.7935522325718669	0.0181021642661009
0.6266793759794507	0.7404877746856744	0.0064922098447312
0.7714560384024658	0.8008102359467201	0.2389597875917778
0.7269882367454089	0.8309862476788964	0.3012523688627798
0.9334972044144634	0.7130406844458697	0.6583044098747032

0.9682669523589669	0.7367576776441662	0.7392032448371576
0.0767492877149699	0.1926612600154147	0.5803337660365473
0.0688336969816044	0.2692834552994084	0.5765592409386888
0.0770692674262935	0.1426655806953013	0.8896849583410446
0.0270158661357691	0.1329285693723452	0.8255525708400776
0.1176462282734919	0.1818647385495210	0.1334945884609069
0.0815400908860662	0.1505959038050307	0.2110988040913831
0.8828423543873535	0.6807727337509587	0.0471184129473840
0.8575530292203418	0.6373632432875753	0.9734162681256747
0.0924928697799978	0.8125118205563345	0.9184592603240384
0.0547335803936126	0.8734707548197510	0.9203626517870466
0.7864925010090507	0.1256926090286970	0.3226939307257723
0.7831831581606554	0.1676967783701490	0.2325073550110001
0.2181488681323666	0.7344617271102651	0.8607338811252017
0.1767858445784453	0.6791964115696698	0.8851419774526853
0.9702142108103691	0.7246639694521916	0.1086480945433556
0.9483666057706751	0.6565770032377091	0.1411380173019721
0.9423047950664343	0.3837843936646149	0.9447433478302555
0.9612107181987513	0.4082926807477638	0.0382797091267712
0.8876428889691059	0.8701860776672937	0.8377271163637406
0.9178506068120550	0.8900530213523575	0.7480738012913393
0.7965629201473390	0.5699475283180327	0.1702797783184116
0.8339593502014903	0.6042660930910871	0.0998628490338198
0.9378391605050340	0.6439014895360811	0.3159208114728075
0.9317240342260384	0.6376249383004513	0.4175410740772965
0.9994262119874878	0.0527949351578062	0.7454498133138635
0.9863639931120800	0.1191435692344022	0.6976054318866425
0.1127349737815015	0.0113041764070418	0.2291773103397938
0.1272000228868193	0.0703344901226084	0.1706916028528848
0.2885035024704790	0.3763676633819824	0.9297454309195915
0.3077890964896918	0.4481439390727219	0.9067322579401167
0.1249732243153589	0.3512010308477251	0.6694175798363227
0.1223260161806884	0.3717909827915331	0.7682370465252729
0.8577510491873898	0.7884092520633933	0.2692029707123497
0.8419859807369255	0.8566944067919102	0.2423722558056902
0.2660201868974337	0.8298581407061993	0.8693562356746175
0.2076733282883545	0.8317344413748694	0.8223579488969208
0.3800456916326714	0.2042147729670493	0.8649507668393208
0.3502406917836617	0.2356706708222153	0.9458396333059805
0.9149234381532388	0.8317954893427101	0.3832727148108193
0.9073250924873857	0.8997103985842366	0.3451634337754808
0.7166344331452272	0.8328931679470422	0.1424525512312652
0.7495507178967704	0.8209378774279877	0.0542492703419628
0.9485783859126039	0.0759686302081699	0.3307270694064935
0.9633335100227659	0.0510520922696736	0.4241629242704357
0.2332303195638221	0.3699909327466767	0.1392474554042852
0.2306620943131018	0.3180983159567374	0.2124247986538785
0.8852080141967934	0.2272473248987040	0.4967368934924983
0.9099332783936138	0.2184514047895418	0.5932838368889031
0.5298790776422239	0.8996733587822818	0.3373675745676017
0.5480980094398931	0.8415958703680746	0.3968365962809971

0.2633539351239919	0.0951157047127489	0.9323775505316070
0.3137713877944511	0.1451535801257702	0.9065267359702834
0.9946642603584869	0.2097375375685426	0.6262113315706598
0.9621308241134852	0.2189878259140847	0.7158210155979434
0.4371458972269567	0.8548623317733594	0.9646399694291672
0.5028584271462845	0.8467677561246846	0.9403216331578812
0.3623995638133268	0.0467381095794180	0.3930574972152328
0.4090359580801065	0.0979608385648697	0.3689262230806568
0.3310793535320225	0.6535041055456875	0.8500001637184450
0.2648174243456104	0.6460203979616057	0.8461318642871568
0.1135143087508240	0.5398324370885683	0.3649838829698775
0.0583013385843290	0.5182681050325844	0.3157623951645039
0.3160840246341912	0.8918242866634202	0.7972637502320389
0.3549022772845725	0.8330484922821670	0.8254475842843353
0.0285441288285160	0.9960049538988244	0.3106620232439353
0.0506410024590827	0.9271702289175531	0.2838741547461726
0.9454203627123262	0.3150531310989545	0.0607483108166010
0.9682300045611092	0.2439173870490086	0.0760771772279384
0.1919892123091246	0.0599903949214950	0.2727602864832659
0.2331668282157260	0.1161242407980572	0.3094843173641262
0.1829866691301438	0.5264812042487577	0.5293399148203909
0.1612244902763335	0.4573563794422492	0.5248132219810250
0.7928496075864975	0.0611556670246910	0.2090009940427515
0.8112495043395228	0.0047064165056559	0.1437135650824816
0.6002048678605785	0.6603569312913128	0.1504186247832525
0.5842490170063206	0.6062763054076933	0.2145900004292598
0.8756743135992701	0.3756771483286651	0.6210314008218077
0.8894584682602409	0.3169415010543447	0.5615915509291192
0.1890110176836286	0.1598055550073740	0.7350047840726163
0.2189203206456085	0.0943383545023073	0.7222028278849678
0.9546068782513014	0.1109257609687856	0.0779379527662937
0.9663277330722202	0.0420267945734514	0.1166087675337003
0.0696447559228628	0.0967645865365735	0.6402915610266440
0.1342246518055849	0.1099383721377486	0.6482934882230807
0.7075318288014032	0.7243494737244350	0.2829945585073248
0.6618661842431398	0.6709896982058029	0.2698804154339648
0.7910348392524099	0.9026324082657392	0.6059083305833461
0.7993440607023194	0.8398436883529080	0.5461575511309097
0.7468690891911360	0.0568379542785229	0.3996619679154270
0.7538729764052063	0.1108812656273551	0.4696302858578513
0.0910611280314818	0.3005353882803229	0.1078752578483484
0.0257913417986146	0.3071572722713661	0.1331927168838261
0.1172159172431874	0.7456664408984055	0.1371729265893365
0.1142789128044806	0.7247259151821280	0.0386248407542272
0.9116346593094894	0.3186345726522605	0.2247897550166597
0.9008215622756168	0.2887697086567882	0.3171419761966213
0.8449112414354970	0.5050245170837717	0.0568716147298650
0.8768464477474640	0.4486215778852044	0.0084932046990555
0.2100103640633567	0.8363201869937906	0.9809756262779986
0.1726362567508976	0.8148263624987482	0.0605095757966589
0.0230547020775255	0.8339566061969517	0.2398906280764722

0.9988154424142491	0.8582433030669741	0.3285142150856634
0.0256003883603340	0.4928151217260416	0.1709696004035297
0.0577658994102528	0.5565508793847471	0.1855670510207191
0.8267117927120274	0.9253401459585732	0.7368834414713616
0.8043513221211208	0.9859150099236079	0.6915949747185181
0.8680659365843546	0.9087039144132961	0.5387602679864840
0.9217962157051947	0.9065694980894077	0.4777774695839656
0.9795718021124794	0.6456343440399575	0.9988967487370360
0.9516456623395116	0.6090998584797969	0.9203547098500600
0.3526451859355826	0.7601425020591336	0.1247948809329527
0.4052845127106078	0.7580045580221006	0.1831528326931283
0.1509287535543762	0.4534346317968177	0.8806003404875279
0.2174874275806969	0.4533931240335543	0.8908533374262371
0.7948003225579181	0.5841561249667218	0.7537104881294390
0.8514817913860212	0.6033216922778679	0.7080072462288213
0.2949512163292418	0.3167824544883254	0.6893390457011922
0.3044763688155741	0.2620116801447662	0.6171694699586013
0.3258609516207642	0.3287918380725736	0.3638101873871483
0.3195748219233236	0.3693288130370860	0.4534545709714778
0.9315274833783453	0.2372123581837733	0.8451257331691189
0.9986222404882495	0.2252372263992240	0.8452568148775976
0.4338657444228121	0.2138647901226464	0.7323455720862110
0.3677085715161398	0.2365738170362602	0.7215604720167110
0.1697697265897564	0.1898851768059041	0.5025820707319686
0.1802367573876028	0.2206735586383948	0.5954271341958476
0.8318993792267748	0.2477978803341781	0.6545374479439444
0.7729631098421983	0.2443129402789494	0.7013830463476243
0.7860950681247988	0.2291970942773065	0.1231656164922360
0.7227455577229941	0.2089714940102619	0.1477151913360731
0.7345378984713089	0.0046854873049034	0.2842313517808381
0.6716068021313220	0.0191400432952349	0.3120074598491287
0.8058033014115270	0.9147878358018605	0.3349330093693275
0.8261891773693913	0.9609227345365183	0.2609969490647328
0.3384783812089275	0.8937051614224512	0.4813032889016258
0.3038028401149460	0.9395754448530530	0.4247634034591962
0.6361056672194428	0.0405282848986247	0.1981042673559306
0.5889163841089265	0.0065996819766802	0.2547249237250615
0.2906531676901911	0.9769206901260793	0.7213930391158733
0.3187554201280390	0.9239181612829696	0.6613184550487625
0.8672883485447979	0.5794672996238245	0.8472473913925384
0.8755377577764208	0.5425466172613695	0.9382751272010043
0.0324622072697872	0.4637101662487850	0.0431058214962228
0.0435705902956025	0.4024666107049009	0.1056581214202416
0.8512434278722175	0.7611172204861937	0.4235118901491490
0.9036848927299300	0.7264281009212378	0.3757608228006540
0.4198604149338969	0.7907348565607942	0.7426758193876506
0.4336195018369672	0.7962062706795757	0.8442246138064697
0.9979207571280502	0.4484781018047538	0.5755668708272581
0.9614977597930587	0.3909928967044250	0.6015823039704447
0.2885710926910307	0.0876084244518489	0.6231842868136452
0.2961560702194142	0.1402079683234460	0.5513758181047868

0.0378005640598808	0.2372980286053898	0.4582965188629854
0.0400999730427790	0.2031820486724678	0.3662578355343517
0.8831858484294195	0.9427786823936161	0.0823331550589610
0.8914359252361429	0.0182231042163384	0.0833930941381912
0.0206648236254967	0.5095839989456171	0.4456321433181753
0.9638493198599482	0.4676745709499573	0.4435891053372293
0.3612145140612807	0.5295108879869199	0.9700686602102380
0.3257416033713242	0.5555173022346160	0.8916491306667547
0.9724956141181551	0.8272593366387854	0.1077151314951923
0.0352771423016960	0.7977436104345643	0.0978512831069482
0.0493167191271700	0.3710246512733446	0.5990360359476700
0.0992522136804866	0.3666377262655869	0.5304591431148376
0.8400063503762444	0.6715621060464398	0.6129983759884012
0.8913276650507665	0.6286591249422384	0.5867335009780833
0.8821668406845862	0.8373275788517730	0.1245490390040263
0.8953834796146445	0.8429960137150085	0.0227510664114854
0.0510397080667144	0.4873514324149272	0.9047013645111380
0.0227104668222418	0.5499190566999260	0.9481855657256371
0.8695366030926298	0.1251595122457491	0.2673582367935746
0.9235949773646730	0.1104156152597668	0.2098860323838858
0.3183103995151761	0.8522806922746782	0.1746977134903408
0.2710274386517325	0.8389413963666003	0.1005329007481019
0.1716662696444984	0.9201872703512373	0.0467233416738743
0.1498777101745392	0.9809587542550862	0.0992672105397460
0.5136355131526017	0.8319959944849264	0.1582568116051800
0.5543888946226286	0.8545205393852390	0.0793708243734475
0.8068446529568662	0.0981130319307162	0.6639095269302281
0.8556074571223692	0.0624018941501024	0.6129370091568885
0.2348546319758971	0.1869231612905465	0.9105928082159870
0.2027866179970567	0.2369617887064236	0.8573180792284526
0.4825188508054225	0.8266342569238944	0.3000013628902739
0.4799729940235476	0.7598441255089772	0.2595794241810968
0.1565347880362747	0.8767804309330678	0.7080908662664678
0.2240634155330372	0.8907814286491054	0.7050661476418149
0.1717752942794451	0.0967155451491167	0.4139301486331216
0.1292618958808512	0.1032382850503673	0.4979749226425581
0.7660130647106469	0.2933028794785952	0.2380097924004929
0.8193115499929513	0.3304015867217447	0.2755352359267753
0.2811105308942985	0.0578981874966497	0.1944734485066960
0.3265497516446197	0.0516837029145464	0.1187232695746903
0.4376969144317869	0.7150728598522209	0.0652500718515159
0.4680807686425353	0.7516357762142806	0.9904463531848330
0.9301563525698432	0.3860518890840599	0.7983110064618363
0.9686333450859643	0.3306982924936400	0.8290593827523669
0.8429898165407477	0.2270735122545958	0.8184886640519868
0.8694245324772111	0.1658572118358949	0.8591887119152002
0.7175649399319952	0.0342123496194109	0.1289138807985853
0.6713563355688641	0.0116235530944300	0.0609812559448417
0.0719369313315165	0.8511104439962031	0.7782483251036345
0.0401338653109556	0.8717133927633947	0.6932120598367675
0.2212053957957471	0.3778120367024543	0.7212737426057632

0.2715642273025278	0.3928682677055725	0.7852279392635730
0.7609005769735212	0.9884469516987874	0.8793159885154763
0.7308759205916130	0.9588437692914534	0.8006249530300282
0.0032664871540611	0.7735191946754033	0.8747828224638721
0.9805219628043694	0.7034679064879249	0.8859938667116932
0.1159636942199442	0.3783469234416639	0.3907070154992002
0.1009640654921101	0.4471973334357980	0.4157489635778034
0.9151793191768867	0.1914977416309455	0.3405378683128182
0.9576154785457304	0.2357938250104332	0.3895745367071853
0.1081064995397916	0.5386918555009945	0.0020968424491816
0.1698206563542798	0.5352685646851589	0.9739145196096134
0.7455087589023589	0.7508847049584065	0.9498287081008165
0.8015124930722735	0.7239273655334469	0.9944781498277333
0.8995191717193266	0.3993914199944062	0.4617033106337273
0.9088241661762707	0.3927989134272074	0.3598890356097781
0.4412996861896756	0.0813580145097528	0.7280730318620054
0.3961828455473065	0.1318882818042848	0.7091964559554633
0.8271242085130884	0.4097970750180057	0.7490158466799605
0.8356379878394827	0.3373019958020356	0.7343170719618913
0.8773150165684638	0.5607605539867981	0.4736914230976372
0.9417977610783621	0.5510215756880752	0.4950219054174087
0.6302493287847798	0.1775750901513750	0.1370333266238182
0.6702540985846956	0.1262702325759398	0.0984505805389855
0.1614359964387950	0.0949128112208162	0.9424778731613426
0.1529066788097858	0.0991363240698446	0.8405588035911639
0.2384889544655040	0.2500536224497837	0.7182426208754875
0.1762175447635459	0.2787822351145210	0.7339143261593322
0.2678414785139874	0.0378700738434553	0.3819461545449506
0.2917157087207905	0.0475570120675560	0.4778170146803296
0.2856362995839266	0.3063431041441798	0.0419300832765359
0.2639667097901742	0.2770931856590598	0.9544479045124212
0.6690214058009004	0.4944286453646579	0.7279325474723907
0.5236016464681900	0.6860295356012251	0.3501694053929129
0.4884108720161153	0.6055619411336466	0.3009420580475902
0.7169998930283225	0.4218295669551718	0.6828181961028453
